# Exploring the Involvement of Gut Microbiota in Cancer Therapy-Induced Cardiotoxicity

**DOI:** 10.3390/ijms24087261

**Published:** 2023-04-14

**Authors:** Kunika, Norbert Frey, Ashraf Y. Rangrez

**Affiliations:** 1 Department of Cardiology, Angiology and Pneumology, University Hospital Heidelberg, 69120 Heidelberg, Germany; 2 DZHK (German Centre for Cardiovascular Research), Partner Site Heidelberg/Mannheim, 69120 Heidelberg, Germany

**Keywords:** gut microbiome, dysbiosis, cancer treatment, cardiotoxicity

## Abstract

Trillions of microbes in the human intestinal tract, including bacteria, viruses, fungi, and protozoa, are collectively referred to as the gut microbiome. Recent technological developments have led to a significant increase in our understanding of the human microbiome. It has been discovered that the microbiome affects both health and the progression of diseases, including cancer and heart disease. Several studies have indicated that the gut microbiota may serve as a potential target in cancer therapy modulation, by enhancing the effectiveness of chemotherapy and/or immunotherapy. Moreover, altered microbiome composition has been linked to the long-term effects of cancer therapy; for example, the deleterious effects of chemotherapy on microbial diversity can, in turn, lead to acute dysbiosis and serious gastrointestinal toxicity. Specifically, the relationship between the microbiome and cardiac diseases in cancer patients following therapy is poorly understood. In this article, we provide a summary of the role of the microbiome in cancer treatment, while also speculating on a potential connection between treatment-related microbial changes and cardiotoxicity. Through a brief review of the literature, we further explore which bacterial families or genera were differentially affected in cancer treatment and cardiac disease. A deeper understanding of the link between the gut microbiome and cardiotoxicity caused by cancer treatment may help lower the risk of this critical and potentially fatal side effect.

## 1. Introduction

Over the past several decades, tremendous efforts have been made to develop a range of treatment options for cancer, including chemotherapy, radiotherapy, immunotherapy, and surgery, all of which have significantly reduced the mortality and morbidity associated with various forms of cancer [1]. Although the benefits of anticancer drugs and therapies are undeniable, safety aspects cannot be overlooked; for example, some treatments have been found to adversely affect the cardiovascular system [2,3]. Cardiotoxicity is a crucial factor to consider for individuals receiving cancer therapy because, even though most patients will recover from cancer, they will then have elevated long-term cardiac risks [4,5]. However, in-depth insights into the underlying molecular mechanisms and causal agents are lacking. Interestingly, a pioneering study including 1526 tumors and their adjacent normal tissues across seven cancer types (breast, lung, ovary, pancreas, melanoma, bone, and brain tumors) reported a distinct microbiome composition specific to cancer types [6]. The authors also correlated bacteria identified in the tumor microenvironment (TME), or their predicted functions, with tumor types and subtypes, smoking status, and response to immunotherapy [6]. Similarly, one of the long-term effects of cancer treatment is a shift in the composition of the gut microbiome, termed gut dysbiosis [7]. Importantly, low-grade chronic inflammation is one of the hallmarks of cardiac diseases and cancers, and is also caused by gut dysbiosis and an altered intestinal permeability barrier. Can the gut microbiome be the missing link? Well-directed and thorough research is required to answer these questions.

The microbiome is defined as a collection of all microbial genomes found in a particular environment. Rapid advances in tools and techniques in recent years have provided new knowledge and insights into the interactions between microorganisms and their hosts [8]. Humans and microbes have evolved together, and microbial communities play a significant role in maintaining human health [9]. The number of gut microbiota, the microbial commensal organisms of the gastrointestinal system, exceeds the number of human cells and constitutes the largest surface area for microbial interactions with the host’s immune system. In contrast, alterations in the microbial composition of the gut, or gut dysbiosis, play important physiological roles, primarily promoting the accumulation of proinflammatory substances [9]. Moreover, the gut microbiome plays an important role in regulating the risk of several chronic diseases, including inflammatory bowel disease, obesity, type 2 diabetes, cardiovascular disease, and cancer [9]. Of additional interest, it has been demonstrated that the gut microbiota co-evolves with the host and lies at the intersection of multiple antitumor and oncogenic metabolic, immune, and inflammatory pathways in cancer [10]. Several studies have highlighted that the connection among gut microbiota, genotoxins, and inflammatory responses to microbiota is associated with carcinogenesis [11]. Along these lines, it has been shown that the gut microbiota can vary the host response to chemotherapy through various mechanisms, including immune interactions, xenometabolism, and alterations in community structure [12]. These and similar findings put forth and support the gut microbiome–cancer axis, and a better understanding of these complex interactions may lead to new and better cancer therapy approaches [13,14].

**Figure 1:**
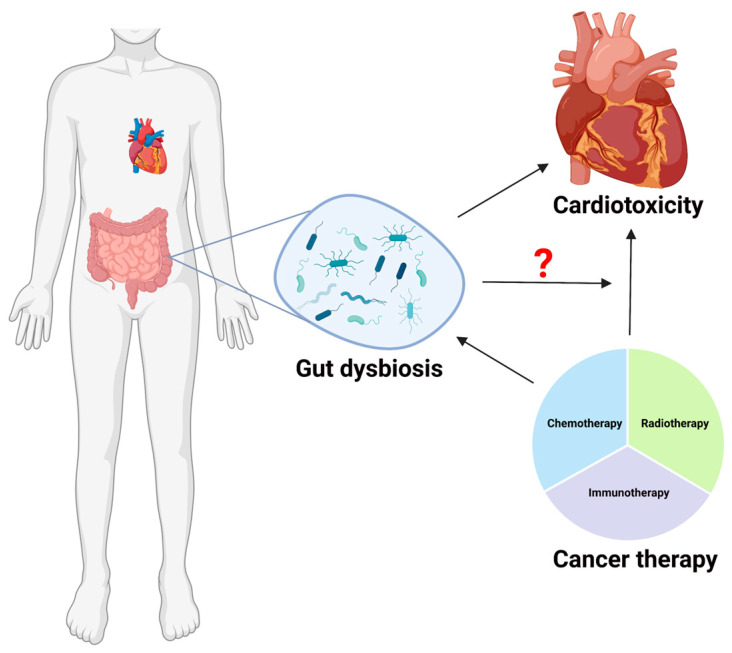
Among the most severe late side effects of cancer treatment in cancer survivors are cardiovascular problems, including heart failure, myocardial ischemia, hypertension, thrombosis, and arrhythmias. Based on the current literature, we know that cancer therapy could result in gut dysbiosis. On the other hand, it is also recently reported that gut dysbiosis could worsen the cardiac function. However, we still do not know if and how gut dysbiosis play a role in cancer therapy induced cardiotoxicity (as shown with a question mark in the pictorial representation). Large-scale cancer survivor microbiome research may help identify patients at cardiovascular risk who may benefit from a more specialized microbiota-mediated treatment. Additionally, a deeper understanding of the link between the gut microbiome and cardiotoxicity brought on by cancer treatment may make it possible to lower the likelihood of this major and fatal adverse effect. (This Schematic representation was created using Biorender (https://biorender.com/)).

In this review, we provide an overview of the potential relationship between gut microbiota and cancer treatment-induced cardiovascular toxicity (Figure 1). We also briefly discuss microbial signatures that are either unique or distinctly regulated in cancer therapy and heart diseases. Although thorough and targeted research is lacking in this direction, based on the available data on the microbiome in cancer therapy, we postulate a plausible relationship between treatment-induced microbial changes and long-term effects via the microbiota–gut–heart axis.

## 2. Cancer Treatment Efficacy and Toxicity Are Influenced by Gut Microbiota

Over the past century, tremendous progress has been made in cancer treatment, resulting in improved quality of life and survival for cancer patients. However, these advances in cancer treatment are often accompanied by treatment-related complications, including secondary systemic side effects. Recently, there has been increasing evidence that cancer treatments can disrupt the host immune response, leading to gut dysbiosis, disturbed immune system, and reduced effectiveness of the treatment [15,16]. Several studies have shown that the absence of gut microbiota reduces therapeutic efficacy, suggesting that commensal microbes modulate treatment-induced anticancer immune responses through various mechanisms. Ground-breaking results from animal models demonstrated the importance of commensals in regulating the effectiveness of radiation therapy, chemotherapy, and immunotherapy drugs [17,18,19]. For example, the intestinal microbiota and the myeloid cells that infiltrate tumors while a patient is receiving platinum-based therapy have been linked together [17]. The current understanding of the relationships among gut bacteria, host reactions, and anticancer medication was analyzed by Huang et al. in 2022 [20], with an emphasis on the immunomodulatory function of microbiota, which supports the effectiveness of immune checkpoint inhibitors. Importantly, this work focuses on the intricate and dynamic relationships between the microbiota and anticancer drugs, as well as the possibility for microbiome-based therapies to potentially enhance the effectiveness of cancer treatment. Huang and colleagues discovered that the gut microbiota affects the pharmacokinetics and pharmacodynamics of anticancer drugs. Furthermore, their work emphasizes the possibility of using microbiota-based therapies, such as probiotics or fecal microbiota transplantation, to increase the efficacy and decrease the toxicity of anticancer medications, as well as the response to immunotherapy. Additionally, anticancer medication response and individualized cancer treatment may be predicted using microbiota-based biomarkers. Nevertheless, additional systematic studies are required in order to completely comprehend the complex connections between the microbiota and anticancer therapies and determine the best methods for modifying the microbiota in order to improve cancer treatment outcomes.

Studying the relationship between microbes and cancer will contribute to a better understanding of the role of microbes in the mechanisms underlying tumorigenesis and other types of cancer, and hopefully improve therapeutic efficacy [21]. Sivan et al. [22] showed that the microbiome could be modulated to alter cancer immunotherapy. An important finding of this study was that *Bifidobacterium* alone could enhance tumor control to a level comparable to that of programmed cell death protein 1 ligand 1 (PD-L1) specific antibody therapy (checkpoint blockage) [22]. Antibodies targeting cytotoxic T-lymphocyte-associated antigen 4 (CTLA-4) have been effective in cancer immunotherapy, and specific *Bacteroides* species are required for the anticancer effects of CTLA-4 inhibition [23].

T-cell responses specific to *B. thetaiotaomicron* and *B. fragilis* have been linked to the effectiveness of CTLA-4 inhibition in mice and humans [23]. Nevertheless, thorough research is required in order to fully understand the mechanisms underlying the effects of the gut microbiota on treatment toxicity.

The non-specificity of several chemotherapeutic agents causes damage to healthy, non-cancerous cells [24]. Chemotherapy-related toxicities include cardiovascular and metabolic diseases, secondary cancer, avascular necrosis, cognitive impairment, cancer-related fatigue, poor mental health-related quality of life, nephrotoxicity, hypogonadism, neurotoxicity, pulmonary toxicity, anxiety, and depression. Cardiotoxicity caused by chemotherapy results in severe heart dysfunction, with heart failure (HF) as the most serious outcome. Following radiotherapy or chemotherapy, there is a considerable risk of developing cardiovascular disease (CVD), particularly in individuals with breast cancer and hematological malignancies [25]. Fibrosis, vascular damage, and shrinkage of damaged tissues or organs are examples of chronic toxicities caused by radiotherapy [26]. Although there is a dearth of information about whether and how the microbiota controls the response to radiotherapy, investigations of drug–microbiome interactions have revealed that several chemotherapeutic agents, such as gemcitabine, vidarabine, and etoposide phosphate, have impaired therapeutic efficacy, reduced mouse survival, and increased cytotoxicity [27]. Similarly, according to a previous study [28] pelvic radiation therapy altered the gut microbiota, with a 10% decrease in *Firmicutes* and a 3% increase in *Fusobacterium*. Through several mechanisms, including modulation of immunological responses, the gut microbiota has been shown to influence the efficacy and toxicity of several chemotherapies and immunotherapies [14]. Thus, targeting the microbiota is a potential strategy for increasing the effectiveness of chemotherapy and decreasing its toxicity.

Taken together, these results suggest that the efficacy of cancer therapy and the degree of gastrointestinal toxicity caused by cancer therapy are influenced by gut microbiota. Studies have indicated that gut microbes play an important role in cancer therapy by reversing anticancer effects and modulating the efficacy of drugs that mediate toxicity. These gut microbes may provide new avenues to improve the efficacy, reduce the toxicity of current chemotherapeutic agents, and improve susceptibility to immunotherapy.

### 2.1. Gut Microbiota and Immunotherapy

Immunotherapy with anti-PD-1/PD-L1 is more successful in tumors with inflamed T cells than in tumors with insufficient T cells because the PD-1/PD-L1 axis is known to play a crucial role in regulating immune system function. Recent research has indicated that gut microbiota may influence the PD-1/PD-L1 axis and the development of innate and adaptive immune systems [22,29]. According to a univariate study, gut microbiota diversity, *Faecalibacterium* abundance, and *Bacteroidetes* diversity were the best indicators of immunotherapy effectiveness. The effect of *Faecalibacterium* on the treatment response was proven by the FMT of responders and non-responders to anti-PD-1 in mice. One study also discovered that the biggest predictor of response to anti-PD-1 medication was the ratio of advantageous to non-beneficial operational taxonomic units (OTUs) [30]. Patients with a baseline majority of *Faecalibacterium* and other *Firmicutes* had longer Progression Free Survival (PFS) than those with a baseline predominance of Bacteroidetes, according to a study by Chaput et al. that analyzed the feces of 26 melanoma patients receiving ipilimumab [31]. Zheng et al. demonstrated a connection between certain gut flora and the effectiveness of immunotherapy in the treatment of liver cancer. The study discovered that *Akkermansia* and *Ruminococcus* were more prevalent in the gut microbiota of responders in hepatocellular carcinoma patients following PD-1 inhibitor therapy [32].

### 2.2. Gut Microbiota and Chemotherapy

Galloway-Pea et al. found a steady decline in the overall microbial diversity over time in the microbiota of AML patients after chemotherapy [33]. They observed an increase in Lactobacillus and a decrease in the anaerobic species *Blautia* [33]. Remarkably, chemotherapy increased the incidence of intestinal domination, a condition in which more than 30% of intestinal bacteria originate from a single taxon. The majority of the dominant incidents were caused by opportunistic pathogenic bacteria, such as *Staphylococcus*, *Enterobacter*, and *Escherichia* [33]. Deng et al. also compared the fecal microbiota composition of 33 healthy controls and 14 colorectal cancer (CRC) patients receiving tegafur and oxaliplatin [34]. Only CRC patients had *Veillonella dispar* in their systems, as opposed to healthy controls. *Prevotella copri* and *Bacteroides plebeius* were also enriched in chemotherapy patients compared with controls [34]. Another study (*n* = 43) of CRC patients with stages II–IV reported increased ratios of *Bacteroidetes* to *Firmicutes*, *Bacteroidetes*, *Bilophila Comamonas*, *Collinsella*, *Butyricimonas*, *Eggerthella*, and *Anaerostipes*, and decreased ratios of *Morganella*, *Pyramidobacter*, *Proteus*, and *Escherichia-Shigella* following CTX [35]. Diversity and composition of the gut microbiome were compared using feces from patients (*n* = 28) before and after CTX [36]. At the genus level, *Ruminococcus*, *Oscillospira*, *Blautia*, *Lachnospira*, *Roseburia*, *Dorea*, *Coprococcus*, *Anaerostipes*, *Clostridium*, *Collin-sella*, *Adlercreutzia*, and *Bifidobacterium* are significantly less common [36]. To date, there is a paucity of literature elucidating the specific mechanisms by which the gut microbiota may contribute to the development of cardiovascular disease in patients who have undergone chemotherapy, and it is an important limitation of this review. Although some studies have indicated a potential link between changes in the gut microbiome and cardiovascular risk factors, such as inflammation and insulin resistance [37], the exact pathways underlying these changes remain poorly understood. Chemotherapy can cause negative effects on the heart, commonly known as chemotherapy-induced cardiotoxicity, which may lead to various clinical symptoms, such as reduced ejection fraction, cardiac arrhythmias, hypertension, and ischemia/myocardial infarction [38]. These cardiotoxic effects can have a significant negative impact on the quality of life and outcomes of cancer patients. Although several categories of chemotherapy agents have been associated with an increased risk of cardiotoxicity, the underlying mechanisms are not yet fully elucidated. Identifying patients at high risk of cardiotoxicity before treatment and monitoring them closely during and after therapy are critical measures in minimizing the effects of chemotherapy-induced cardiotoxicity on patient outcomes. On the other hand, recent studies suggest that the gut microbiota can indirectly influence the development of chemotherapy-induced cardiotoxicity, through the production of metabolites and other signaling molecules [39]. For instance, specific bacteria in the gut can produce compounds (such as butyrate) that interact with the immune system and modify the expression of genes involved in cardiac function and repair [40]. A growing body of evidence indicates that the gut microbiome plays a significant role in protecting against chemotherapy-induced bloodstream infections. Montassier et al. proposed a microbiota-based predictive risk index model that could potentially be utilized to stratify patients at risk of complications before treatment [41]. This model is based on the observation that microbiome diversity decreases before the commencement of therapy. Moreover, the gut microbiota can influence the metabolism of chemotherapy drugs, which could result in increased toxicity or altered efficacy. For example, Wallace et al. showed that gastrointestinal biota can metabolize the chemotherapy drug irinotecan into a toxic by-product, which, in turn, can cause severe diarrhea in some patients [42]. The researchers identified a bacterial enzyme, beta-glucuronidase, responsible for this process, and demonstrated that inhibiting the enzyme reduced the toxicity of irinotecan in mice [42]. These findings indicate that the microbiota can influence the metabolism of chemotherapy drugs, which may result in increased toxicity or altered efficacy.

Trimethylamine N-oxide (TMAO) is a metabolite that is produced by certain gut bacteria from dietary nutrients, such as choline and carnitine [43]. Chemotherapy-induced changes in the gut microbiota can increase the production of TMAO, which has been linked to an increased risk of atherosclerosis and cardiovascular disease. In addition, TMAO can also be implicated in chemotherapy-induced cardiotoxicity by exacerbating the negative effects of chemotherapy on the cardiovascular system. Research has shown that the composition of gut microbiota can affect the production of TMAO, and that certain gut bacteria are more efficient at producing TMAO than others [44]. Chemotherapy can cause changes in the gut microbiota, leading to an increase in the abundance of bacteria that produce TMAO. This increase in TMAO production can contribute to the development of cardiovascular disease, and may also exacerbate chemotherapy-induced cardiotoxicity [45]. Thus, gut metabolites such as TMAO might serve as a link between gut microbiota-induced cardiotoxicity and chemotherapy-induced cardiotoxicity. In addition, an imbalance in gut microbiota composition and function can lead to chronic inflammation, oxidative stress, and other factors that can contribute to the development of cardiovascular disease, including cardiotoxicity induced by chemotherapy [43,46]. While the exact mechanisms underlying the relationship between gut microbiota and chemotherapy-induced cardiotoxicity are not yet fully understood, these findings suggest that targeting the microbiome may be a promising strategy for mitigating the cardiovascular side effects of cancer treatment. However, more research is required in order to gain a better understanding of the complex interactions among the gut microbiota, cancer therapies, and cardiovascular health.

### 2.3. Gut Microbiota and Radiotherapy

Radiotherapy results in dysregulation of the gut microbiota, which negatively affects the diversity and richness of gut bacterial diversity, potentially causing an enrichment of harmful microbiota (*Proteobacteria* and *Fusobacteria*) and a decrease in beneficial microbiota (*Faecalibacterium* and *Bifidobacterium*) [47,48]. El Alam et al. discovered a significant alteration in the gut microbiome composition during pelvic chemotherapy and radiotherapy (CRT), with increases in *Proteobacteria* and decreases in *Clostridiales*, whereas after CRT, the gut microbiome composition changed, with increases in *Bacteroides* species [49]. Intestinal radiation injury is a disorder that can be influenced by radiotherapy, by altering bacteria that produce short-chain fatty acids (SCFAs) [48]. Uncertainty persists regarding the effect of SCFAs on the prevalence of various disorders.

### 2.4. Gut Microbiota and TME

The gut microbiota shapes the immune system in the early years of life, and alterations in the gut microbiota later in life have a significant impact on numerous immune system functions [50]. The relationship between the gut microbiota and the host immune system increases the likelihood that the TME will interact with larger systemic microbial–immune networks, which serves as a reminder that the gut microbiota is increasingly acting as a crucial TME regulator [51,52,53,54,55]. The intestinal bacterium *B. pseudolongum* produces the metabolite inosine, which, by acting on the adenosine A2A receptor on T cells, significantly promotes Th1 cell differentiation in the presence of exogenous IFN-γ and improves the therapeutic response to immune checkpoint inhibitor (ICI) therapy, including anti-CTLA-4 and anti-PD-L1 [56]. A study showed that some gut bacteria, including *Bacteroides* and *Ruminococcaceae*, can contribute to the development of hepatocellular carcinoma by escalating hepatocyte inflammation, building up toxic substances, and causing liver steatosis [57]. According to the “holobiont” idea, it was recently proposed that the gut microbiome influences the “TME”, which in turn affects tumor growth [52,58]. Ohtani et al. reported that the intestinal microbiota of obese individuals increases the amount of deoxycholic acid in the blood, which in turn promotes liver carcinogenesis by causing hepatic stellate cells to exhibit a senescence-associated secretory phenotype [59,60]. Altogether, these findings strongly suggest the crucial role that gut dysbiosis plays in the influence on TME microbiota and the efficacy of cancer therapeutic drugs/modes.

## 3. Cancer Treatment-Induced Cardiovascular Toxicity

The two major causes of death worldwide, accounting for approximately 50% of all deaths, are cancer and cardiovascular disease [61]. Recent cancer treatment strategies have improved patient survival rates. Nonetheless, many cancer therapies have undesired deleterious side effects on the cardiovascular system [62,63]. For example, breast cancer survivors have been shown to be at a significantly higher risk of death due to cardiovascular disease, outweighing the mortality risk of the original cancer or its recurrence [64]. Through an interdisciplinary approach involving cardiologists and oncologists, the cardio-oncology field is working to develop optimal strategies for patients with cardiovascular disease or risk factors from cancer diagnosis throughout the rest of their lives, even after treatment ends. Heart failure, myocardial ischemia, and myocardial infarction are just a few of the conditions that fall under the umbrella term “cardiotoxicity”, which also covers a wide range of other conditions. Increasing therapeutic effectiveness has increased cancer patient survival; however, the long-term cardiovascular effects of these therapies have gained clinical significance. With more than 3.5 million breast cancer survivors in the US, both conventional chemotherapy (such as anthracyclines and radiotherapy) and targeted medicines (such as HER2 inhibitors and CDK4/6 inhibitors) have significantly improved patient care. Since then, cardiovascular disease has overtaken other conditions as the main killer and morbidity factor in this group [65,66]. Owing to their well-known cardiovascular side effects and comparatively high incidence of heart failure, anthracyclines have been the most extensively researched medication for decades [67]. Hoffmann et al. [68] reported that doxorubicin and trastuzumab treatment of nude mice in an orthotopic mouse model of human breast cancer led to a cardiovascular defect. In order to effectively treat cancer, new strategies are urgently needed to prevent potential cardiovascular diseases. Changes may occur years after therapy is over, and may be abrupt or persistent [69].

## 4. Gut Microbiota and Cardiovascular Toxicity

Heart failure has long been associated with impaired intestinal barrier function, which leads to gut dysbiosis and bacterial translocation [70,71,72]. Interestingly, the gut microbiome is increasingly reported to influence cancer development and progression in different ways [73]; for example, on one hand, several types of cancers result in altered gut microbiota, whereas the efficacy of cancer therapies, chemo- and immunotherapy, for example, is found to be strongly influenced by microbiome composition [74,75]. The chemo–gut study, a cross-sectional survey exploring physical, mental, and gastrointestinal health outcomes in cancer survivors, has recently provided novel insights into the strong association between chemotherapy and chronic, moderate-to-severe gastrointestinal symptoms lasting for years after cancer treatment, which are associated with worse mental and physical health [76,77]. Thus, is the gut microbiome a common link between cancer therapy and cardiotoxicity? The answer to this question is still not clear, due to the lack of concrete data on the relationship among microbiota, vascular damage, and heart failure in cancer patients after therapy; however, a few recent studies have suggested that the postulated link is not far-fetched. For example, Huang et al. [78] and Liu et al. [79] have recently shown that an imbalance in the gut microbiome composition and its functional alterations are likely to be among the major etiological mechanisms underlying doxorubicin-induced cardiotoxicity. Importantly, Huang et al. [78] showed that the gut dysbiosis due to doxorubicin contributes to the development of cardiotoxicity, by altering doxorubicin metabolism and increasing inflammation. Furthermore, they observed improved cardiac function and reduced doxorubicin-induced cardiotoxicity upon microbial depletion with the use of antibiotics. Overall, these results strongly suggests that the gut microbiota may potentially serve as new therapeutic target for cardiotoxicity and cardiovascular diseases. Similarly, Zhao et al. [80] observed that cisplatin, one of the chemotherapy drugs that is known to cause cardiotoxicity, led to a dramatic reduction in *Firmicutes* and elevated levels of pathogenic bacteria. On the other hand, *Lactobacillus* supplementation in cisplatin-treated mice increased body weight, improved cardiac function, and attenuated inflammation. The study thus showed that probiotics may help avoid cardiotoxicity brought on by chemotherapy, but additional validation studies are required in order to establish the best probiotic strain, dosage, and duration for this usage.

Similarly, Lin et al. demonstrated that yellow wine polyphenolic compounds protect against doxorubicin-induced cardiotoxicity by modulating the composition and metabolic function of gut microbiota [81]. Thus, it will not be surprising if researchers in the near future consider the gut microbiota as a new target for the treatment of cardiotoxicity and cardiovascular diseases. Large-scale cancer survivor microbiome investigations may help identify individuals at cardiovascular risk who could benefit from more specialized microbiome-mediated treatment. Furthermore, a deeper understanding of the connection between gut microbiota and cardiotoxicity caused by cancer therapies may pave the way for lowering the risk of these grave and potentially deadly side effects.

### 4.1. Gut Microbiota and Heart Failure

Heart failure (HF) is a serious health problem that negatively affects mortality and morbidity worldwide [82]. The levels of several proinflammatory cytokines in plasma are correlated with the severity and prognosis of the disease in patients with heart failure, who are thought to experience a persistent systemic inflammatory response [83,84]. The gut is a blood-demanding organ, and because of its restricted blood supply, the villi (and microvilli) are vulnerable to functional ischemia. A drop in the pH of the intestinal mucosa can result in intestinal ischemia in patients with HF [85]. A decline in intestinal mucosal pH is an indicator of intestinal ischemia in patients with HF [86]. Gut microbiota composition and metabolic parameters of patients with chronic heart failure (CHF) were significantly different from those of the control group, according to a fecal metagenomic study of 53 patients with chronic heart failure and 41 control participants [87]. Patients with HF are almost invariably found to have impaired intestinal barriers [88]. *Yersinia enterocolitica*, *Candida*, *Campylobacter*, *Salmonella*, *Shigella*, and other pathogenic bacteria are more prevalent in patients with CHF than in healthy controls [89]. According to the NYHA scale, these changes are strongly associated with HF severity [89].

### 4.2. Gut Microbiota and Atherosclerosis

Atherosclerosis is a chronic inflammatory condition characterized by a lipid core and an outer fibrous cap that mostly affects the middle and major arteries. A substantial cause of mortality, atherosclerosis is an immunoinflammatory condition that results in blockages in the large and medium arteries and acute CVD [90]. The presence of bacteria in the atherosclerotic plaques of patients with coronary artery disease was confirmed by fluorescence in situ hybridization and conserved polymerase chain reaction [91]. Macrogenomic sequencing of the feces of the subjects was performed in a case-control study of 218 patients with atherosclerotic cardiovascular disease (ACVD) and 187 healthy controls. This study found increased copy numbers of bacterial genes encoding TMA lytic enzymes (enzymes associated with TMAO production), increased TMAO production, and increased abundance of the atherosclerotic cardiovascular disease (ACVD) gut microbiome comprising *Enterobacteriaceae* and *Streptococcus*, among other significant metabolic alterations functionally associated with ASCVD [92]. New approaches for the identification and management of atherosclerosis may emerge from the study of gut microbiota and its metabolites. Dietary factors and gut flora are strongly linked to the development of atherosclerosis, with inflammation also playing a role. For instance, an increase in *Bacteroides fragilis* resulted in a decrease in *Lactobacilli* and an increase in *Desulfovibrionaceae*, which caused dysfunctional lipid or glucose metabolism and worsened the inflammatory response [93]. Peanut skin extract reduced the serum total and low-density lipoprotein cholesterol content, and increased the high-density lipoprotein cholesterol content in atherosclerotic mice, thereby decreasing the development of atheromatous plaques [94].

**Figure 2:**
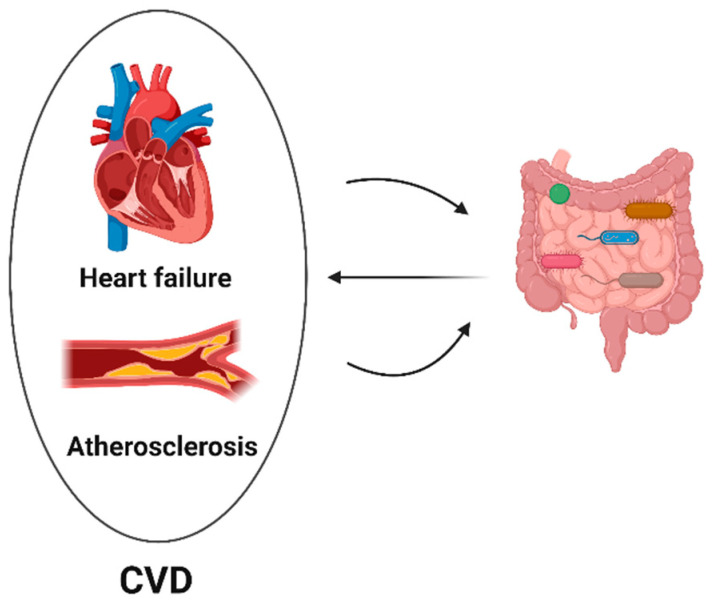
Crosstalk between cardiovascular disease and gut microbiota. Various microbes in the gut are represented with different colors. (This Schematic representation was created using Biorender (https://biorender.com/)).

Dysbiotic intestinal flora can worsen cardiovascular diseases. Through altered gut microbiota composition, immune cell activation, and metabolic dysfunction, an imbalance in the gut microbiota caused by poor diet, aging, and antibiotic usage might exacerbate cardiovascular diseases. Disruption of the gut microbiota may, in turn, be further promoted by cardiovascular disorders (Figure 2). A balanced gut microbiota may prevent the progression of CVD.

## 5. Microbial Signatures Associated with Cancer Treatment and Cardiac Diseases

The most prevalent bacterial phyla in a healthy gut microbiome are *Firmicutes* and *Bacteroidetes*, followed by *Proteobacteria*, *Actinobacteria*, *Fusobacteria*, and *Verrucomicrobia* [95]. *Prevotella* spp. and *Bacteroides* spp. are the most prominent *Bacteroidetes* members, whereas *Bifidobacterium* is the most significant *Actinobacteria* representative [96]. However, gut microbial composition is dramatically altered in several cardiovascular diseases and distinct forms of cancer (Table 1). Interestingly, gut microbial alterations associated with the progression or pathogenicity of many of the cardiovascular diseases have similarly been reported in many cancer therapies, suggesting a potential link between cancer therapy-induced cardiotoxicity and gut dysbiosis. Thus, it is important to understand whether and what kind of correlations exist among cancer therapies, gut dysbiosis, and cardiovascular diseases.

Types of bacteria in cancer treatment:

Some cancer chemotherapies, radiotherapy, and immunotherapies have been reported to be affected by the gut microbiota, in terms of both their effectiveness and toxicity. Chemotherapeutic treatments used to treat cancer cause gut dysbiosis, which is followed by a decline in commensal microorganisms, such as *Bifidobacterium* and *Lactobacillus*, and an increase in opportunistic pathogens, such as *Clostridium difficile* [96]. By controlling the immune system, the intestinal microbiota can affect the therapeutic efficacy of medications for tumors. In contrast to non-responders (NR), PD-1 responders (R) with lung cancer in both Europe and the US had a higher relative abundance of *Akkermansia muciniphila* [97]. Studies also revealed a tendency toward a higher frequency of *Corynebacterium aurimucosum* and *Staphylococcus haemolyticus* in NR patients and a higher frequency of *Enterococcus hirae* in R patients [98]. According to some reports, Fluorouracil (5-FU) therapy results in dysbiosis in mice. After 5-FU administration, the abundance of *Bacteroides* and *Lactobacillus* species decreased, whereas that of *Staphylococcus* and *Clostridium* species increased [99]. Compared to controls, fecal samples from 36 juvenile leukemia patients receiving high-dose methotrexate chemotherapy and 36 healthy children showed a substantial decrease in *Bifidobacterium*, *Lactobacillus*, and *Escherichia coli* [100]. The intestinal barrier is broken and intestinal crypts undergo apoptosis as a result of radiotherapy [101]. In a small pilot study, radiotherapy plus antibiotics reduced *Firmicute* abundance and increased *Proteobacteria* abundance in three pediatric cancer patients with pelvic rhabdomyosarcoma [102].

Types of bacteria in cardiac diseases:

Recent studies have highlighted a possible contribution of the gut microbiome to CVD. CVDs is linked to a higher *Firmicutes*/*Bacteroidetes* (F/B) proportion. The phylum *Bacteroidetes* was found to be negatively associated with ischemic heart disease (IHD), and the order *Lactobacillales* was positively associated with IHD in a small case-control study (*n* = 128) [98]. Type 2 diabetes mellitus, a key CVD risk factor, has been sparsely linked to *Acidaminococcus*, *Aggregatibacter*, *Anaerostipes*, *Blautia*, *Desulfovibrio*, *Dorea*, and *Faecalibacterium* [103]. The reduced abundance of bacteria that produce short-chain fatty acids (SCFAs), such as *Roseburia*, *Faecalibacterium*, and *Eubacterium rectale*, and an increased abundance of host opportunistic pathogens, such as *Escherichia coli*, *Clostridium ramosum*, *Bacteroides caccae*, and *Eggerthella lenta* have been linked to a higher risk of CVDs [104,105]. Researchers have found that, compared to healthy controls, patients with atherosclerosis have reduced relative abundances of *Roseburia* and *Eubacterium* and greater relative abundances of *Collinsella* [106]. Intestinal mucosal barrier degradation and dysbacteriosis caused by lower cardiac output in HF are accompanied by elevated levels of pathogenic bacteria, such as *Candida*, and decreased levels of anti-inflammatory bacteria, such as *Faecalibacterium prausnitzii*. Additionally, elevated levels of some gut bacterial species, such as *Escherichia coli*, *Klebsiella pneumonia*, and *Streptococcus viridans* have been linked to heart failure [107].

Altogether, gut bacteria, unique or common to cancer treatment and cardiac diseases, offer a new avenue for research to learn more about clinical outcomes, potential treatments, and diagnosis, as well as a better understanding of the role of microbes in the development of cancer treatment-induced cardiotoxicity. Gut microbiota can be used as a biomarker to predict the effects of cancer therapy. Populations at high risk may receive more specialized treatment, depending on their microbiota compared to a generic one.

## 6. Gut Microbiota-Derived Metabolites in Cancer Treatment

**Figure 3:**
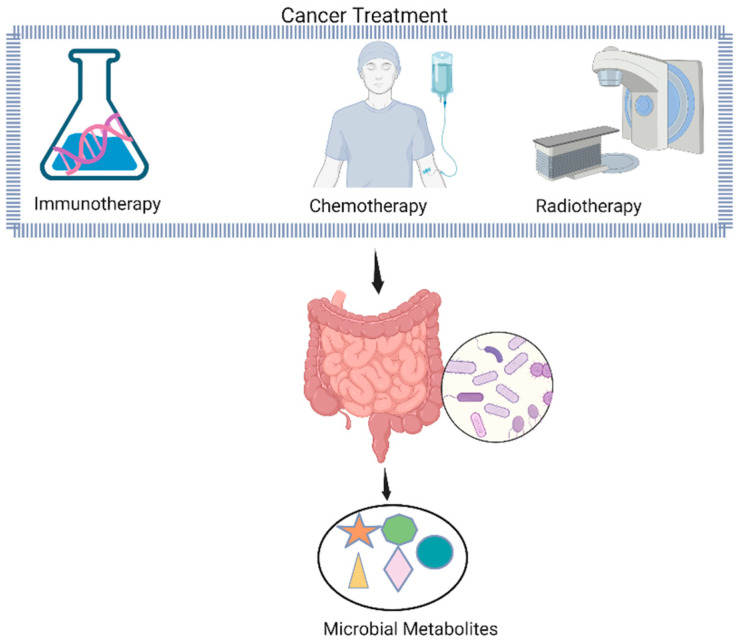
Metabolites produced by the gut microbiota have a key role in controlling the activity of intestinal cells, as well as local and systemic immunological and inflammatory responses. The most effective method for dealing with gut microbiota-derived metabolites and their widespread impacts in order to enhance cancer therapy outcomes will need to be determined. Different colors in circle represent different gut microbes, whereas, different shapes in oval form represent individual metabolite, e.g., star shape for metabolite A, triangle for metabolite B, etc. (This Schematic representation was created using Biorender (https://biorender.com/)).

Numerous metabolic illnesses, such as obesity, type 2 diabetes, nonalcoholic fatty liver disease, and cardiovascular disease, are influenced by the gut microbiome and its metabolites. The maintenance of host physiology depends on communication between microbes and their hosts, which is mediated by metabolites generated by the microbiota [108]. These metabolites have been found to affect both the toxicity and effectiveness of cancer treatment, through modulation of immune processes and protective epithelial functions, respectively (Figure 3). Metabolites such as SCFAs, secondary bile acids, polyamines, lipids, and vitamins are produced by gut microbiota [109]. The colon produces SCFAs, primarily acetate, butyrate, and propionate, from dietary fiber and polysaccharides. The most common bacterial species that produce SCFAs include *Faecalibacterium prausnitzii*, *Clostridium leptum*, *Eubacterium rectale*, and *Roseburia* species, as well as lactate-utilizing species such as *Anaerostipes* and *Eubacterium hallii,* which synthesize SCFAs from lactate and acetate [110]. The regulation of T cell homeostasis has been associated with SCFAs that can control the differentiation of T cells into effector or regulatory (Treg) cells in response to immunological conditions, such as the presence or absence of important cytokines [72] Gut microbial metabolites, including bacteriocins, short-chain fatty acids, and phenylpropanoid-derived metabolites, display direct and indirect anticancer activities via different molecular mechanisms [111]. Recent studies have confirmed the differential expression of SCFA in immunotherapy responders compared to non-responders. SCFAs are well known for their anti-inflammatory and antioxidant effects on the host, which help stop the proliferation of cancer cells. Bile acid profiles may change as a result of bacterial bile acid transformation, which may then affect systemic inflammatory and fibrotic processes [72]. In terms of cancer development and anticancer activity, the mechanisms of the action of gut microbial metabolites are not fully understood.

## 7. Conclusions

Patients with cancer endure a variety of immediate and long-term side effects throughout the body, including gastrointestinal- and cardiotoxicity. Preclinical and clinical research, in addition to reports on the link between microbiota and cancer, has revealed that this subject may be a key mediator of how the body reacts to cancer treatment. Clinical trials on a substantial cohort of cancer survivors are urgently needed and may open up novel possibilities for microbiota-mediated therapies to stop or lessen the long-term side effects of cancer therapy. Future therapies may employ techniques that can help achieve more precise manipulation of microbiota composition, such as the relative proportion of a particular bacterial genus in the microbiota. In order to discover dysbiotic conditions linked to negative or poor cancer treatment outcomes and to identify microbial targets that can be modified, personalized biomarkers are urgently needed. Improving the physical well-being of cancer survivors requires a thorough understanding of the microbiota–gut–heart axis and the effects of the changed intestinal microbiome on immunological and metabolic pathways. We can only maximize the regulation of the intestinal microbiota and enhance the potential of cancer treatment by fully comprehending which intestinal bacteria and their metabolic product(s) could be altered. Overall, our review aimed to shed light on the potential complex interplay among the microbiome, cancer treatment, and cardiovascular health, and to identify potential avenues for future research into this important area of study. However, direct evidence supporting proposed postulations and hypotheses is still missing. This lack of significant direct evidence suggests that further research is needed in order to explore the potential effects of gut microbiota and its metabolites in cardiotoxicity, which may lead to new therapeutic opportunities and the identification of predictive biomarkers.

## Figures and Tables

**Table 1:** Some of the gut bacteria associated with cardiac diseases and cancer treatment.

Cancer Treatment	Cardiac Diseases
*Bacteroides fragilis*, *Helicobacter pylori*, *Salmonella tyhimunum*, *Burkholderia cepacian*, *Akkerman-sia muciniphila*, *Faecalibacterium prausnitzii and Bifidobacterium longum*, *Brevundii monas and Staphylococci*, *Firmicutes and Actinobacteria*, *Lactobacillus*, *and Escherichia coli*, *Clostridium difficile*, *Faecalisbacterium*, *and Burkholderia cepacian*, *Aristipes shahi*, *Burkholderia cepacian*, *Akkermansia*, *and Alistipes*	*Bacteroidetes* *Lactobacillales* *Candida* *Faecalibacterium prausnitzii* *Roseburia intestinalis* and *Faecalibacterium* cf. *prausnitzii*

## Data Availability

Not applicable.
